# Circulating adult stem and progenitor cell numbers—can results be trusted?

**DOI:** 10.1186/s13287-019-1403-x

**Published:** 2019-10-17

**Authors:** Julia M. Kröpfl, Michelle Schmid, Yvonne Di Marzio, Karine Schreiber, Christina M. Spengler

**Affiliations:** 1 Exercise Physiology Lab, Institute of Human Movement Sciences and Sport, ETH Zurich, Winterthurerstrasse 190, CH-8057 Zurich, Switzerland; 2 Division of Hematology, University Hospital Zurich, Rämistrasse 100, CH-8091 Zurich, Switzerland; 3 Zurich Center for Integrative Human Physiology (ZIHP), University of Zurich, Winterthurerstrasse 190, CH-8057 Zurich, Switzerland

**Keywords:** Density gradient centrifugation, Red blood cell lysis, Lymphocyte-to-monocyte ratio, Hematopoietic progenitor cell, CD34+/CD45dim

## Abstract

**Background:**

Within the last years, the interest in physical exercise as non-invasive stimulus influencing circulating hematopoietic stem and progenitor cell (CPC) concentrations has constantly grown. Cell estimates are often derived by determining the subgroup of CPC as percent lymphocytes (LYM) or mononuclear cells (MNC) via flow cytometry and back calculation over whole blood (WB) cell counts. However, results might depend on the used cell isolation technique and/or gating strategy. We aimed to investigate MNC loss and apoptosis during the flow cytometry sample preparation process preceded by either density gradient centrifugation (DGC) or red blood cell lysis (RBCL) and the potential difference between results derived from back calculation at different stages of cell isolation and from WB.

**Methods:**

Human blood was subjected to DGC and RBCL. Samples were stained for flow cytometry analysis of CPC (CD34+/CD45dim) and apoptosis analysis (Annexin V) of MNC and CPC subsets. MNC and LYM gating strategies were compared.

**Results:**

Both DGC as well as RBCL yielded comparable CPC concentrations independent of the gating strategy when back calculated over WB values. However, cell loss and apoptosis differed between techniques, where after DGC LYM, and monocyte (MONO) concentrations significantly decreased (*p* < 0.01 and *p* < 0.05, respectively), while after RBCL LYM concentrations significantly decreased (*p* < 0.05) and MONO concentrations increased (*p* < 0.001). LYM apoptosis was comparable between techniques, but MONO apoptosis was higher after DGC than RBCL (*p* < 0.001).

**Conclusions:**

Investigated MNC counts (LYM/MONO ratio) after cell isolation and staining did not always mimic WB conditions. Thus, final CPC results should be corrected accordingly, especially when reporting live CPC concentrations after DGC; otherwise, the CPC regenerative potential in circulation could be biased. This is of high importance in the context of non-invasively induced CPC mobilization such as by acute physical exercise, since these cell changes are small and conclusions drawn from published results might affect further applications of physical exercise as non-invasive therapy.

## Background

The influence of acute physical exercise on circulating hematopoietic stem and progenitor cell (CPC) concentrations has become an intensely researched topic within the last years [1]. The emerging questions range from basic science investigations of the underlying mechanisms on how acute exercise [2–6] or exercise training [7, 8] would influence the circulating progenitor cell number, to the possible applications of exercise as a non-invasive stimulus for regeneration after cardiac incidences [9] and for immature immune cell mobilization [10], or as adjuvant therapy for peripheral artery disease [11]. However, results of different studies are difficult to compare due to differences in exercise protocols, immunological markers and flow cytometry analyses, cell isolation techniques, and gating strategies [12].

Usually, CPC concentrations are estimated by multiplying the percentage of cells of interest of a flow cytometry-acquired cell count (CPC proportion) by circulating cell concentrations of the mature immune system (dual-platform approach). Estimated results are then given as cells per volume whole blood (WB). Although the current gold standard for CD34+ analysis is single-platform flow cytometry using internal reference counting beads or volumetric counting [13], most analyses are done by the dual-platform approach since this method is equally suitable to assess the CPC number [14]. In addition, most flow cytometers do not provide any volumetric information. Two of the few flow cytometers that do include volumetric data are the Attune™ Nxt [15, 16] or the MACSQuant [17]. Machines such as the BD FACSCalibur™ [3] or BD FACSCanto™ II [18], however, only report cell proportions.

Flow cytometry results do not only depend on the choice of a flow cytometer, they also depend on the preceding cell isolation technique and/or gating strategy. CPC content is often reported as proportion of mononuclear cells (MNC)—namely lymphocytes (LYM) and monocytes (MONO)—or as proportion of LYM only, preceded either by density gradient centrifugation (DGC) or red blood cell lysis (RBCL). According to literature, DGC shows from 97 to > 99% red blood cell depletion [19], and even protects LYM from mechanically induced DNA strand breaks [20], but is rather time-consuming. An alternative is RBCL by ammonium chloride solution, which recovers total white blood cells in an easy and fast way [21] and is not supposed to damage (permeabilize) the leucocyte fraction of WB cells [22].

Back calculation to CPC concentrations over the total MNC or LYM count derived from a hemocytometer assumes that no cells are lost or damaged, e.g., driven into apoptosis, during the sample preparation process. More than 20 years ago, Fritsch and coworkers already investigated the loss of nucleated cells after DGC and RBCL and how it would affect WB 34+ numbers [23]. However, the authors did not control results by differential blood smear, the amount of cell loss by the density gradient medium alone, the extent of MNC or CPC apoptosis, or a possible change in the ratio of LYM/MONO. These could all affect the final results.

Therefore, we wanted to investigate MNC loss and apoptosis in dual-platform testing preceded by either DGC or RBCL and the potential difference between results derived from back calculation at different stages of cell isolation and WB cell counts.

## Methods

### Cell analyses overview

**Fig. 1:**
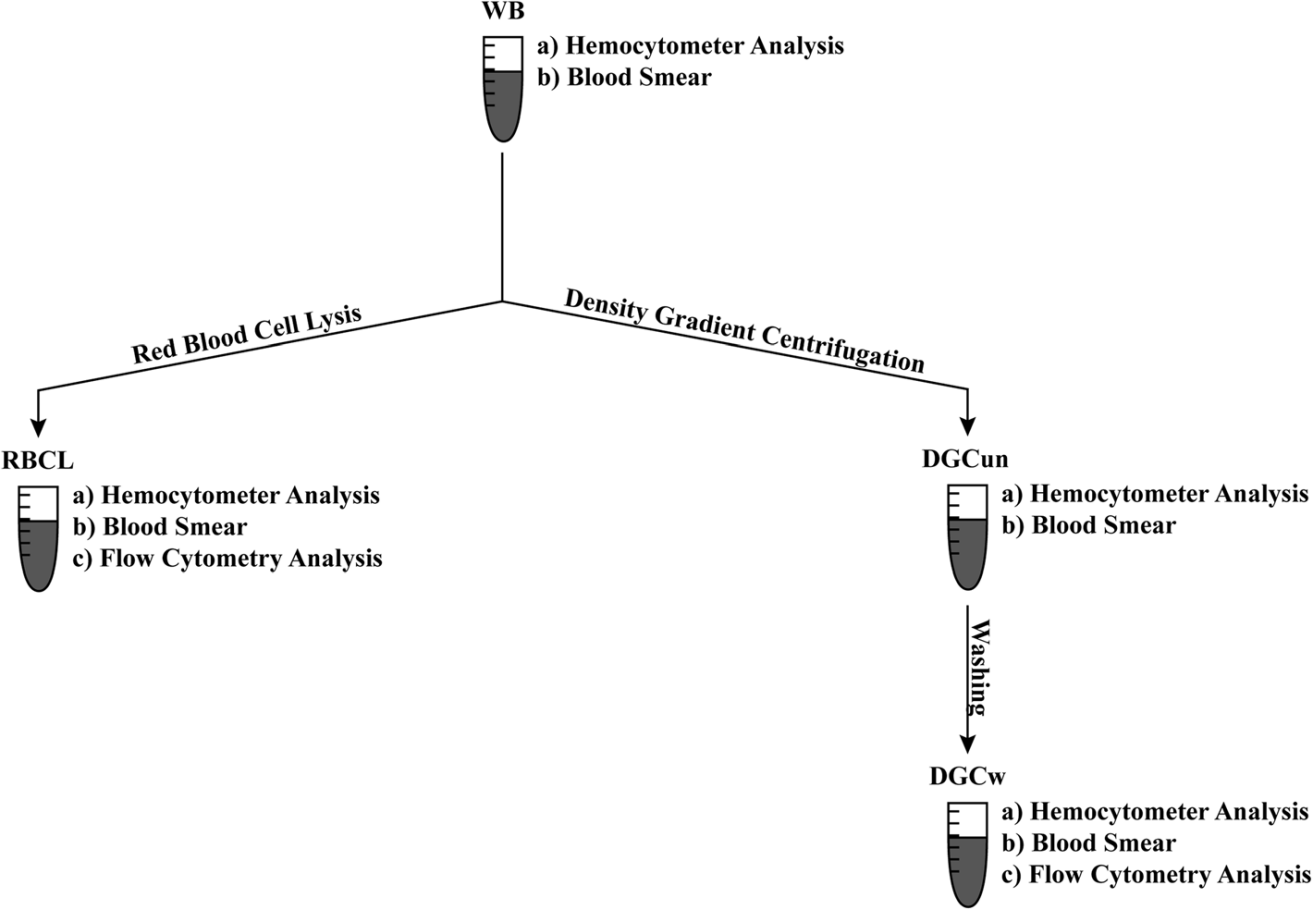
Sample preparation—flow chart. The sample preparation process included (a) hemocytometer analysis, (b) blood smear for each single isolation step, and (c) flow cytometry analysis of samples isolated by density gradient centrifugation and red blood cell lysis. Samples are indicated as follows: whole blood (WB), directly after density gradient centrifugation (DGCun), after density gradient centrifugation, and an additional washing step (DGCw), after the lyse and wash procedure (RBCL)

Fifteen milliliters venous WB was drawn into EDTA tubes. One milliliter WB was used for blood cell count measurements by a hemocytometer (ADVIA 2120i, Siemens, Zurich, Switzerland) [24]. Samples were measured in duplicate except for lysates. Total and differential white blood cell counts were read from the peroxidase channel. If necessary, samples were diluted. Sample preparation for flow cytometry analysis was done in two different ways, first by density gradient centrifugation (DGC) and second using red blood cell lysis (RBCL). An overview of sample preparations can be found in Fig. 1.

### Cell isolation by density gradient centrifugation

Four milliliters WB was diluted with 4 ml of PBS (without Mg^2+^ or Ca^2+^; Cantonal Pharmacy, University Hospital Zurich, Switzerland), overlaid on 4 ml of Ficoll (Histopaque®-1077, Sigma-Aldrich, Buchs, Switzerland), and centrifuged (1300 rpm, room temperature, 30 min, low breaks). The mononuclear cell (MNC) fraction was isolated and resuspended in 4 ml of isotonic saline (Bichsel AG, Interlaken, Switzerland). Sample volume before hemocytometer analysis was readjusted to 4 ml to avoid errors by sample dilution/concentration. Of this solution, 1 ml was used for blood cell counting and smear analysis (Fig. 1, DGCun). The rest of the solution was washed with PBS, centrifuged (1300 rpm, 10 °C, 10 min, full breaks) (Fig. 1, DGCw), and subjected to flow cytometry analysis.

### Cell isolation by red blood cell lysis

For RBCL, 10 ml of 1× ammonium chloride solution (10× RBC Lysis buffer, Lucerna-Chem AG, Lucerne, Switzerland) was added to 500 μl of WB (ratio 20:1) and incubated according to the manufacturer’s instructions (10–15 min at room temperature). After incubation, samples were directly washed with PBS (without Mg^2+^ or Ca^2+^, Cantonal Pharmacy, University Hospital Zurich, Switzerland), centrifuged (1500 rpm, 10 °C, 5 min, full breaks), and resuspended in 500 μl isotonic saline (Bichsel AG, Interlaken, Switzerland) in order to avoid errors by sample dilution/concentration. Of this solution, 300 μl was used to measure blood cell counts (Fig. 1, RBCL), 100 μl was used for cytospin and subsequent differential smear analysis, and another 100 µl was used for flow cytometry analysis.

### Flow cytometry analysis

**Fig. 2:**
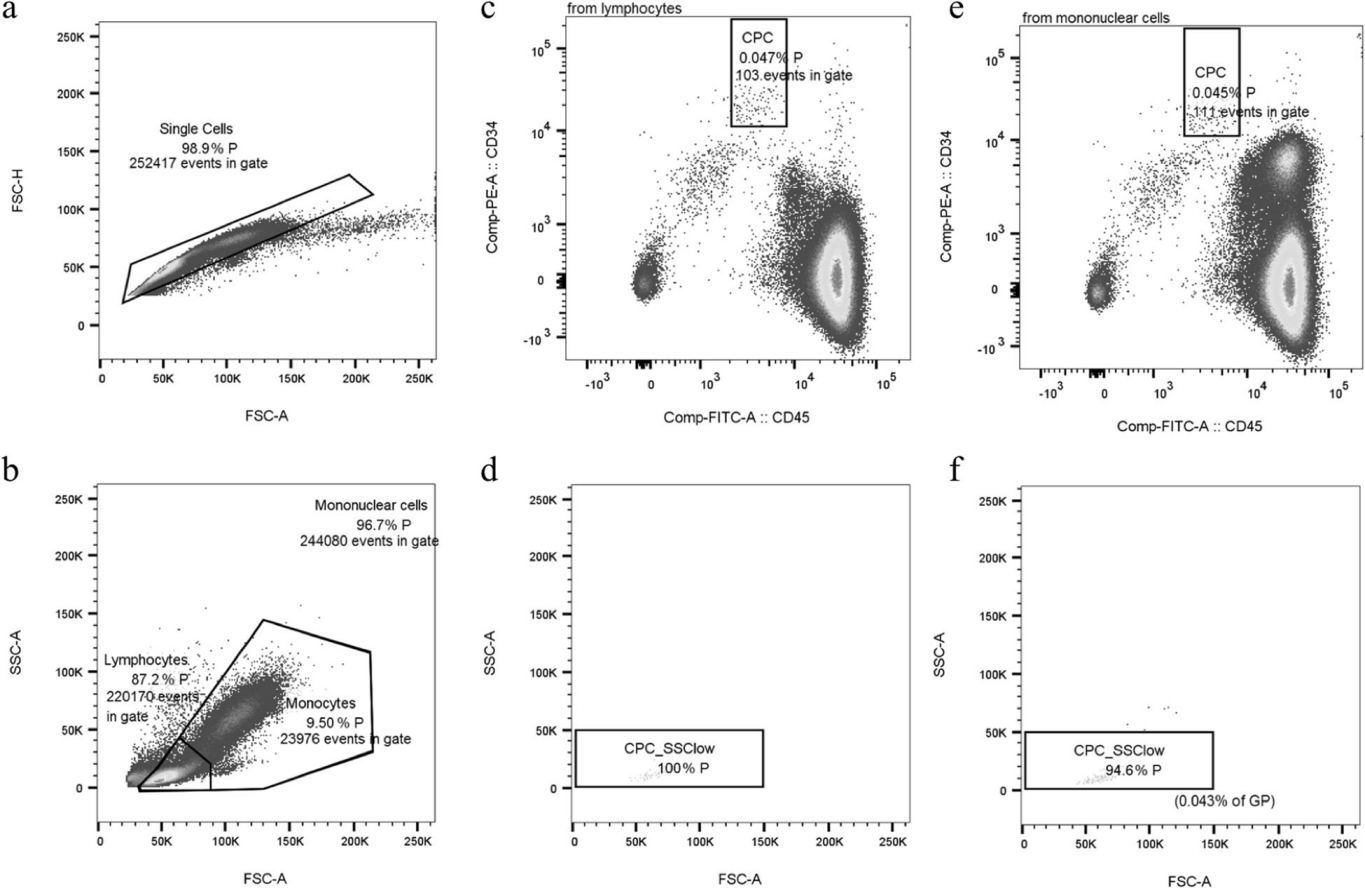
Flow cytometry analysis based on density gradient centrifugation. Gating characteristics (doublet exclusion, **a**; parent (P) populations, **b**) and fluorescent gating (CD34+/CD45dim cells within the lymphocyte gate, **c**; side scatter low, **d**; CD34+/CD45dim cells within the mononuclear cell gate, **e**; and side scatter low within the grandparent population (GP), **f**) after density gradient centrifugation. Absolute numbers represent the absolute amount of cells analyzed in the different gates, while percent numbers indicate cell amount relative to the (grand-) parent population (%P, %GP)

**Fig. 3:**
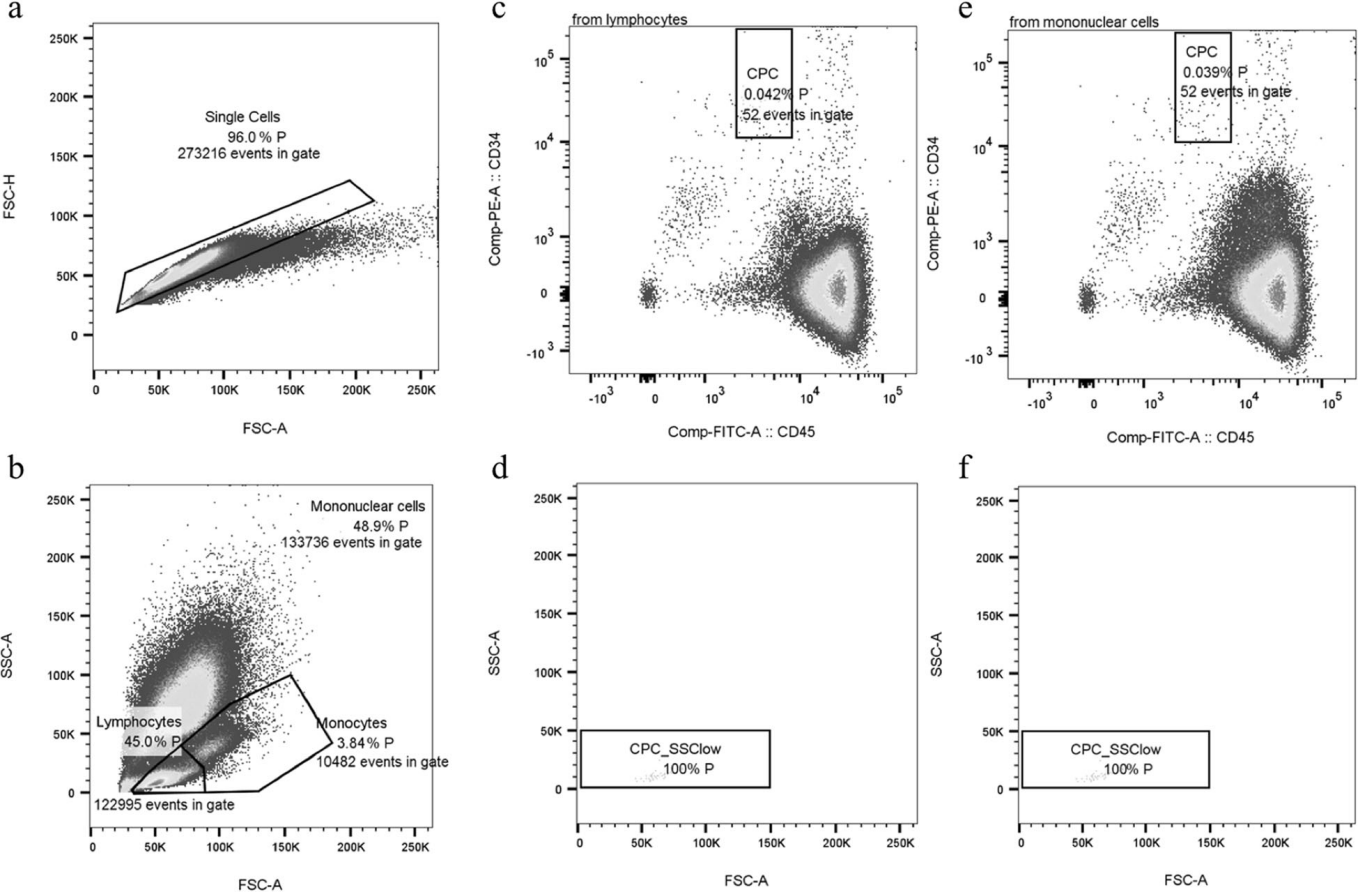
Flow cytometry analysis based on red blood cell lysis. Gating characteristics (doublets exclusion, **a**; parent (P) populations, **b**) and fluorescent gating (CD34+/CD45dim cells within the lymphocyte gate, **c**; side scatter low, **d**; CD34+/CD45dim cells within the mononuclear cell gate, **e**; and side scatter low, **f**) after red blood cell lysis. Absolute numbers represent the absolute amount of cells analyzed in the different gates, while percent numbers indicate cell amount relative to the parent population (%P)

Briefly, 10^6^ MNC were labeled by antibodies CD34-phycoerythrin (PE, clone 4H11, Thermofisher, Schlieren, Switzerland), CD45-fluorescein-isothiocynate (FITC, clone HI30, Thermofisher, Schlieren, Switzerland), and CD31-allophycocyanin-Cy7 (APC-Cy7, clone WM59, Lucerna-Chem AG, Lucerne, Switzerland) and incubated for 30 min on ice in the dark. After incubation, samples were washed and further incubated with a live/dead stain (LIVE/DEAD™ Fixable Aqua Dead Cell Stain Kit, Thermo Fisher Scientific, Zurich, Switzerland) and an apoptosis stain (Annexin V-PerCP-Cy5.5, BD Biosciences, Allschwil, Switzerland) for 15 min at room temperature in the dark. Afterwards, samples were washed and finally fixated with 2% paraformaldehyde in PBS (Fisher Scientific, Ontario, Canada). The fixative was not washed out, but samples were analyzed immediately in order to avoid side scatter loss of granulocytes (GRA) in the RBCL samples due to fixation [25]. Fluorescent minus one samples were used as negative controls. Three-color analysis was performed immediately after staining with compensated fluorescent parameters (BD™ CompBead, BD Biosciences, Allschwil, Switzerland). The acquisition gate was either established based on forward and side scatter characteristics including lymphocytes (LYM), excluding GRA, monocytes (MONO), and debris, or all MNC excluding debris. Hematopoietic stem and progenitor cells (CPC, CD34+/CD45dim) [26, 27] were counted by a FACSCanto2 flow cytometer using the FACSDiva software (BD Biosciences, Allschwil, Switzerland) and a separate analysis tool (FlowJo, LLC, Oregon, USA). Estimates of the CPC concentrations were calculated by multiplying the proportion of each cell subset of the LYM or MNC acquisition gates (DGC Fig. 2c, f and RBCL Fig. 3c, e) by the LYM or the MNC concentrations either in WB, directly after DGC (Fig. 1, DGCun), after DGC and a washing step (Fig. 1, DGCw), or after RBCL and an additional washing step (RBCL).

The analysis of apoptotic MNC and CPC subsets was done by additionally investigating Annexin V and Aqua dot plots and dividing cell populations into four quadrants: early-apoptotic (Q1), late-apoptotic (Q2), necrotic (Q3), and live cells (Q4). This was done for both cell isolation techniques and gating strategies (Additional files 1, 2, and 3).

### Analysis by differential blood smear

Analysis by differential blood smear was done according to standard procedures. Blood was smoothed out, and cell aggregates were prepared by a cytocentrifuge for cell staining and differentiation [28]. May-Gruenwald Giemsa staining provided information on cell morphology.

### Statistics

Data are presented as arithmetic mean and standard deviation. Cell ratios and changes in cell concentrations are presented as individual values or geometric mean. WB values were taken as controls. Variables were tested for normal distribution by the Kolmogorov-Smirnov test. Friedman’s or repeated measures ANOVA were used for comparisons between the different cell isolations steps as well as WB cell proportions, corresponding cell proportions on blood smear and flow cytometry results including Bonferroni post hoc corrections. Related-samples Wilcoxon signed-rank test or paired *t* test was performed to detect differences for investigated parameter proportions and concentrations between DGC and RBCL or between LYM and MNC gating techniques as well as for cell loss and apoptosis between different cell types.

## Results

### Whole blood lymphocyte and monocyte concentrations compared to values after density gradient centrifugation and red blood cell lysis

**Fig. 4:**
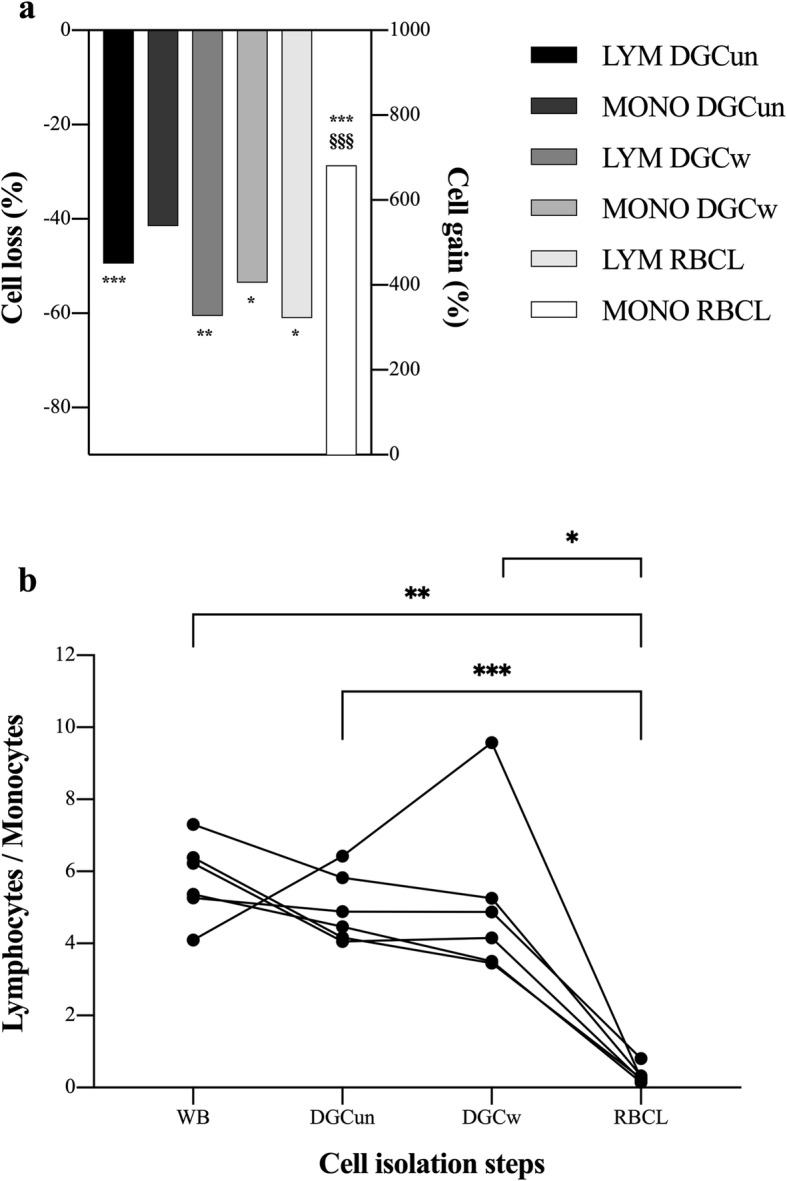
Cell change by the different cell isolation techniques prior to flow cytometry analysis. **a** Lymphocytes (LYM) and monocytes (MONO) were equally affected by the two steps of density gradient centrifugation (DGC): DGC with 1300 rpm for 30 min (DGCun) and an additional washing step with 1300 rpm for 10 min (DGCw). During red blood cell lysis (RBCL), the amount of LYM lost was similar to both DGC steps, but cell gain in MONO seemed to be an artifact due to limitations of the hemocytometer used in this study. Data are displayed as geometric mean. *n* = 6. Repeated-measures ANOVA with Bonferroni post hoc comparisons was used for analysis. Significant losses to whole blood and differences between losses per cell type are indicated as follows: *p < 0.05, **p < 0.01, ***p < 0.001, and ^§§§^*p* < 0.001, respectively. **b** Change of the lymphocyte-to-monocyte ratio between the different cell isolation steps. Individual values are displayed. *n* = 6. Repeated-measures ANOVA with Bonferroni post hoc comparisons was used for analysis. Significant differences are indicated as follows: *p < 0.05, **p < 0.01, ***p < 0.001

Directly after DGC and buffy coat isolation (Fig. 1, DGCun), LYM and MONO concentrations measured by a hemocytometer were decreased by 50% (*p* < 0.001) and 42% (*p* > 0.05) in comparison to WB values, respectively (Fig. 4a). After washing cells with PBS (Fig. 1, DGCw), LYM and MONO concentrations were decreased by 61% (*p* = 0.001) and 54% (*p* = 0.039) in comparison to WB values, respectively (Fig. 4a). LYM and MONO concentrations also differed between DGCun and DGCw samples (*p* = 0.024 and *p* = 0.043, respectively, Table 1). Cell loss did not significantly differ between cell types in DGCun and DGCw samples.

**Table 1:** Hemocytometer and blood smear analyses for the different cell isolation steps

	Whole Blood (WB)	DGC unwashed (DGCun)	DGC washed (DGCw)	RBCL washed (RBCL)
Blood cell counts (hemocytometer)				
WBC, 10^9^/l	5.4 ± 0.8	0.7 ± 0.2***^§§^	0.4 ± 0.1***^‡‡§§^	3.1 ± 0.7***
RBC, 10^12^/l	5.1 ± 0.4	0.0 ± 0.0***	0.0 ± 0.0***	0.0 ± 0.0***
Hct, %	45.4 ± 3.0	0.0 ± 0.0***	0.0 ± 0.0***	0.0 ± 0.0***
Hgb, g/dl	14.9 ± 1.2	0.0 ± 0.0***	0.0 ± 0.0***	0.0 ± 0.0***
HDW, %	23.5 ± 2.0	35.7 ± 28.6	19.3 ± 26.4	28.2 ± 23.2
RDW-CV, %	12.9 ± 0.5	17.9 ± 11.3	18.8 ± 19.4	23.3 ± 26.1
MCV, fl	89.6 ± 3.2	70.5 ± 28.0	43.9 ± 30.6	39.0 ± 32.2
MCH, pg	29.4 ± 1.2	0.0 ± 0.0***	0.0 ± 0.0***	0.0 ± 0.0***
MCHC, g/l	328.2 ± 6.1	0.0 ± 0.0***	0.0 ± 0.0***	0.0 ± 0.0***
Lymphocytes, 10^3^/μl	1.75 ± 0.40	0.89 ± 0.23***	0.70 ± 0.20**^‡^	0.76 ± 0.45*
Monocytes, 10^3^/μl	0.31 ± 0.06	0.18 ± 0.05^§§§^	0.15 ± 0.05*^‡§§§^	2.39 ± 0.39***
Neutrophils, 10^3^/μl	3.1 ± 0.4	0.0 ± 0.0***	0.0 ± 0.0***	1.1 ± 0.7**
Basophils, 10^3^/μl	0.02 ± 0.04	0.00 ± 0.00	0.00 ± 0.00	0.00 ± 0.00
Lymphocytes, %	32.2 ± 3.6	77.5 ± 2.5***^§§§^	76.9 ± 5.3***^§§§^	17.7 ± 11.8
Monocytes, %	5.7 ± 1.1	16.1 ± 2.8**^§§§^	17.0 ± 5.0*^§§^	54.9 ± 6.4***
Neutrophils, %	57.2 ± 4.3	1.5 ± 0.6***^§^	3.0 ± 0.7***^‡§^	24.0 ± 11.4**
Basophils, %	0.6 ± 0.2	0.2 ± 0.1*	0.2 ± 0.2***	0.2 ± 0.1*
Morphological parameters (blood smear)				
Lymphocytes, %	29.8 ± 4.7	77.9 ± 14.8	88.8 ± 3.7++	27.4 ± 7.5
Monocytes, %	7.0 ± 1.7	19.7 ± 15.5	7.3 ± 3.1+	3.3 ± 3.2+++
Rod-shaped granulocytes, %	2.8 ± 2.2	0.6 ± 0.6	0.0 ± 0.0	7.1 ± 6.2
Segmented granulocytes, %	56.3 ± 6.8	0.3 ± 0.3	0.2 ± 0.4	46.0 ± 10.7
Neutrophil granulocytes (rod-shaped + segmented), %	59.2 ± 7.8	0.83 ± 0.82	0.17 ± 0.41++	53.1 ± 9.7+
Basophil granulocytes, %	1.3 ± 1.0	1.3 ± 1.3	1.9 ± 1.4	0.8 ± 0.5
Eosinophil granulocytes, %	2.7 ± 3.1	0.0 ± 0.0	0.0 ± 0.0	2.4 ± 1.7
Debris, %	0.0 ± 0.0	0.0 ± 0.0	1.6 ± 3.9	13.0 ± 4.4

Data are given as arithmetic mean ± standard deviation. *n* = 6; *DGC* density gradient centrifugation, *RBCL* red blood cell lysis, *WBC* white blood cell count, *RBC* red blood cell count, *Hct* hematocrit, *Hb* hemoglobin, *RDW-CV* red blood cell distribution width coefficient of variation, *MCV* mean corpuscular volume, *MCH* mean corpuscular hemoglobin, *MCHC* mean corpuscular hemoglobin concentration; significant differences to WB values and to RBCL are indicated as follows: *p < 0.05, **p < 0.01, ***p < 0.001 and ^**§**^*p* < 0.05, ^**§§**^*p* < 0.01, ^**§§§**^*p* < 0.001, respectively. Significant differences to DGCun are indicated as follows: ^‡^*p* < 0.05, ^‡‡^*p* < 0.01. Significant differences between smear results and respective hemocytometer values are indicated as follows: +*p* < 0.05, ++*p* < 0.01, +++*p* < 0.001

Directly after RBCL, LYM concentration declined by 61% and was significantly lower compared to the control (WB) condition (*p* = 0.029; Fig. 4a), but was comparable to DGCuw and DGCw samples (both *p* > 0.05). MONO concentrations increased by 682% (*p* < 0.001; Fig. 4a) compared to the control condition and significantly differed from both DGCuw and DGCw (both *p* < 0.001, Table 1). Changes in cell concentrations after RBCL significantly differed between cell types (*p* < 0.001).

The ratio of LYM/MONO did not significantly differ between WB (5.7), DGCuw (4.9), and DGCw (4.8) (all *p* > 0.05, Fig. 4b), but was significantly lower in RBCL (0.3, *p* = 0.001).

### Whole blood lymphocyte and monocyte proportions compared to values from morphological analyses by differential smear and flow cytometry

There were no significant differences between the LYM or MONO proportions in WB or DGCun samples and the respective blood smears (Table 1, *p* > 0.05). LYM and MONO proportions in DGCw samples assessed by the hemocytometer (Table 1) were comparable to values found by flow cytometry (Table 2), but LYM proportions were lower and MONO proportions higher than on DGCw smear (*p* = 0.001 and *p* = 0.025, respectively, Table 1).

**Table 2:** Results from flow cytometry analysis

Gated cells as percentage of (grand) parent population	DGC	RBCL
Doublets (%)	1.3 ± 1.1	7.5 ± 2.8**
LYM (%)	82.5 ± 4.4	44.8 ± 4.5***
Live LYM (%)	95.8 ± 0.7	95.3 ± 0.4
Early-apoptotic LYM (%)	4.2 ± 0.7^**§§§**^	4.7 ± 0.4^**§§§**^
Late-apoptotic LYM (%)	0.002 ± 0.000^**§§§**^	0.001 ± 0.000^**§**^
Necrotic LYM (%)	0.006 ± 0.008	0.002 ± 0.002^**§**^
MONO (%)	12.3 ± 2.5	4.6 ± 0.6***
Live MONO (%)	30.4 ± 4.1	62.3 ± 6.1***
Early-apoptotic MONO (%)	69.6 ± 4.1	37.6 ± 6.0***
Late-apoptotic MONO (%)	0.08 ± 0.02	0.05 ± 0.04
Necrotic MONO (%)	0.012 ± 0.007	0.020 ± 0.013
CPC (%LYM)	0.05 ± 0.01	0.06 ± 0.02
CPC (%MNC)	0.05 ± 0.01	0.05 ± 0.02

Data are given as arithmetic mean ± standard deviation. *n* = 6; *DGC* density gradient centrifugation, *RBCL* red blood cell lysis, *LYM* lymphocytes, *MONO* monocytes, *CPC* hematopoietic stem and progenitor cells, *MNC* mononuclear cells; significant differences between cell isolation techniques and between LYM and MONO within the same quadrant and cell isolation technique are indicated as follows: **p < 0.01, ***p < 0.001 and ^**§**^*p* < 0.05, ^**§§§**^*p* < 0.00, respectively

LYM proportions in the RBCL samples were comparable to respective smear results (Table 1), but showed significantly lower values than flow cytometry analysis (*p* = 0.005, Table 2). MONO proportions were significantly higher in the RBCL samples measured by the hemocytometer than on the respective smear (Table 1) or in flow cytometry analysis (both *p* < 0.001, Table 2). Neutrophil GRA (rod-shaped and segmented) proportions were significantly higher on smear than in the RBCL sample detected by the hemocytometer (*p* = 0.012, Table 1).

### Flow cytometry result comparison between samples prepared by density gradient centrifugation and red blood cell lysis

The percentage of doublets was significantly higher after RBCL than after DGC (*p* = 0.004, Table 2).

Both LYM and MONO proportions were enriched after DGC in comparison to RBCL (both *p* < 0.001, Table 2). Neither live, nor early-, late-apoptotic, or necrotic LYM proportions differed between isolation techniques (all *p* > 0.05, Table 2). Live MONO proportions were increased after RBCL in comparison to DGC, while for early-apoptotic MONO proportions it was the contrary (both *p* < 0.001, Table 2). Late-apoptotic and necrotic MONO proportions were comparable between cell isolation techniques (both *p* > 0.05, Table 2). Both early- and late-apoptotic LYM proportions were significantly lower than early- and late apoptotic MONO proportions after both DGC and RBCL, respectively (all *p* < 0.001, except late-apoptotic after RBCL *p* < 0.05, Table 2). Necrotic LYM proportions were also significantly less than necrotic MONO proportions (*p* < 0.05, Table 2) after RBCL.

**Fig. 5:**
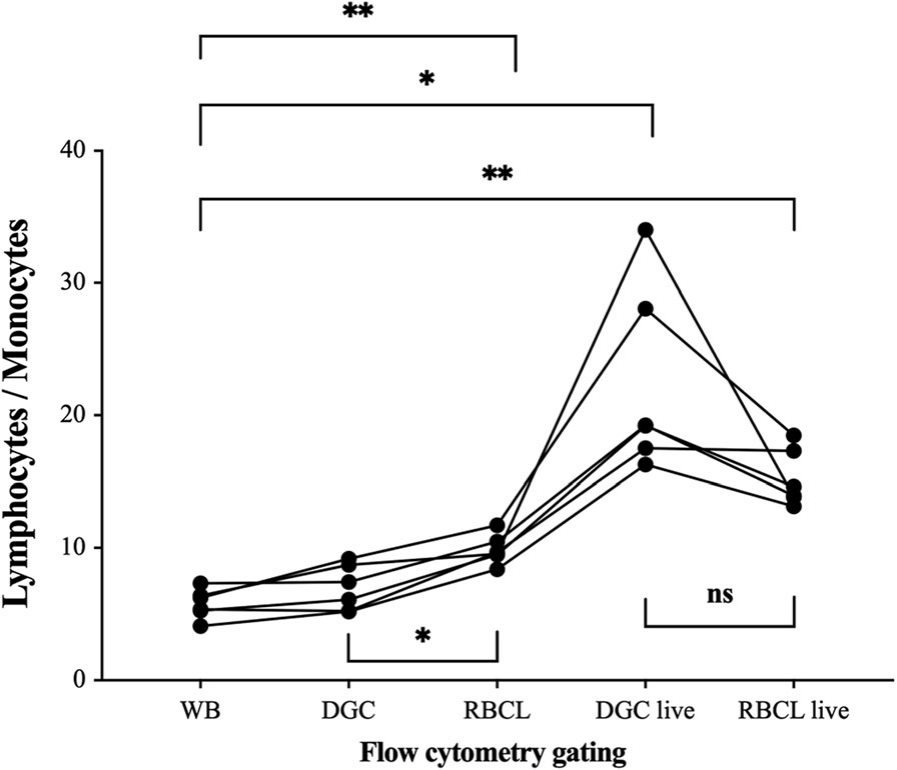
Cell change during flow cytometry analysis. Change of the lymphocyte-to-monocyte ratio as manually gated during flow cytometry analysis. Samples were taken from whole blood (WB), after density gradient centrifugation (DGC), after red blood cell lysis (RBCL), and after identification of live cells by fluorescent gating (DGC live, RBCL live). Individual values are displayed. *n* = 6. Repeated-measures ANOVA with Bonferroni post hoc comparisons was used for analysis. Significant differences are indicated as follows: *p < 0.05, **p < 0.01, ***p < 0.001

The ratio of flow cytometry-counted LYM/MONO was significantly increased after RBCL (9.8) in comparison to WB (5.7, *p* = 0.001) and DGC values (6.8, *p* = 0.018, Fig. 5). The ratios of live LYM/live MONO in the DGC (21.6) and RBCL (15.1) samples were also both significantly increased (*p* = 0.016 and *p* = 0.002, respectively) in comparison to the ratio of LYM/MONO in WB (5.7), but did not differ between isolation techniques (*p* > 0.05, Fig. 5).

**Fig. 6:**
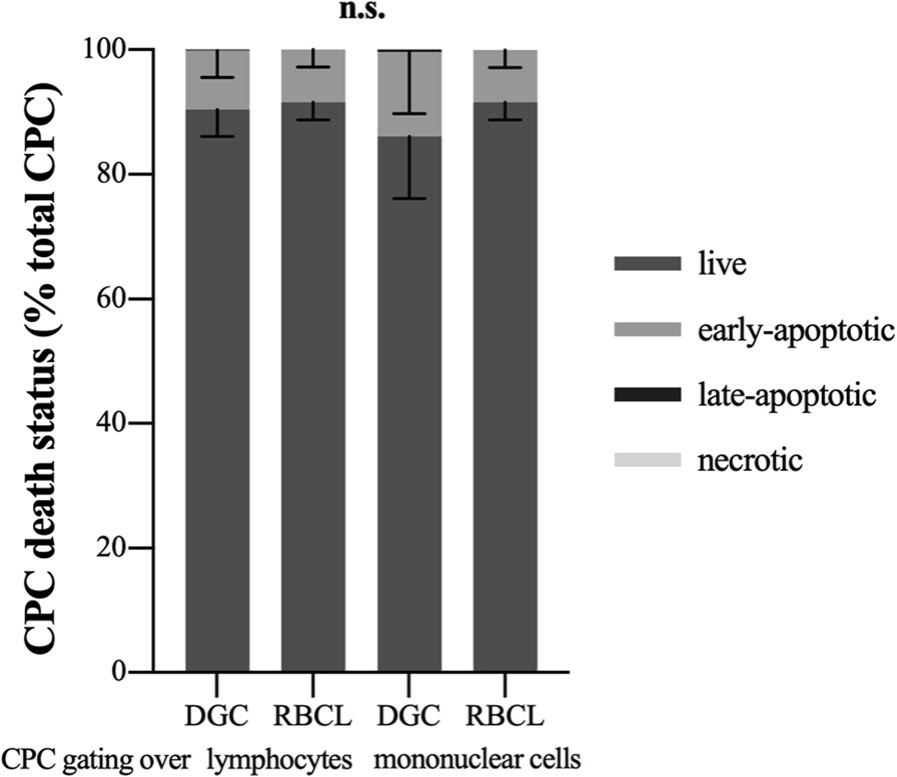
Stem and progenitor cell death status. Live, early-apoptotic, late-apoptotic, and necrotic circulating hematopoietic stem and progenitor cells (CPC) as percentage of total analyzed CPC. Percentages did neither differ between cell isolation—by density gradient centrifugation (DGC) or red blood cell lysis (RBCL)—nor gating over lymphocytes or mononuclear cells. It is to note that live and early-apoptotic CPC make up almost 100% of analyzed CPC. There were only very few late-apoptotic CPC visible after DGC and a complete lack of necrotic CPC. Data are displayed as arithmetic mean and standard deviation. *n* = 6. Repeated-measures ANOVAs with Bonferroni post hoc comparisons were used for analysis. Significant differences are indicated as follows: non-significant, n.s

Total CPC proportions (Table 2) as well as live, early-, late-apoptotic, or necrotic CPC proportions (Fig. 6) detected by flow cytometry did not differ between DGC and RBCL independent of the gating strategy (all *p* > 0.05).

### Back calculation results

Results back calculated over WB cell counts did neither depend on the cell isolation technique nor on the gating strategy (all *p* > 0.05).

**Fig. 7:**
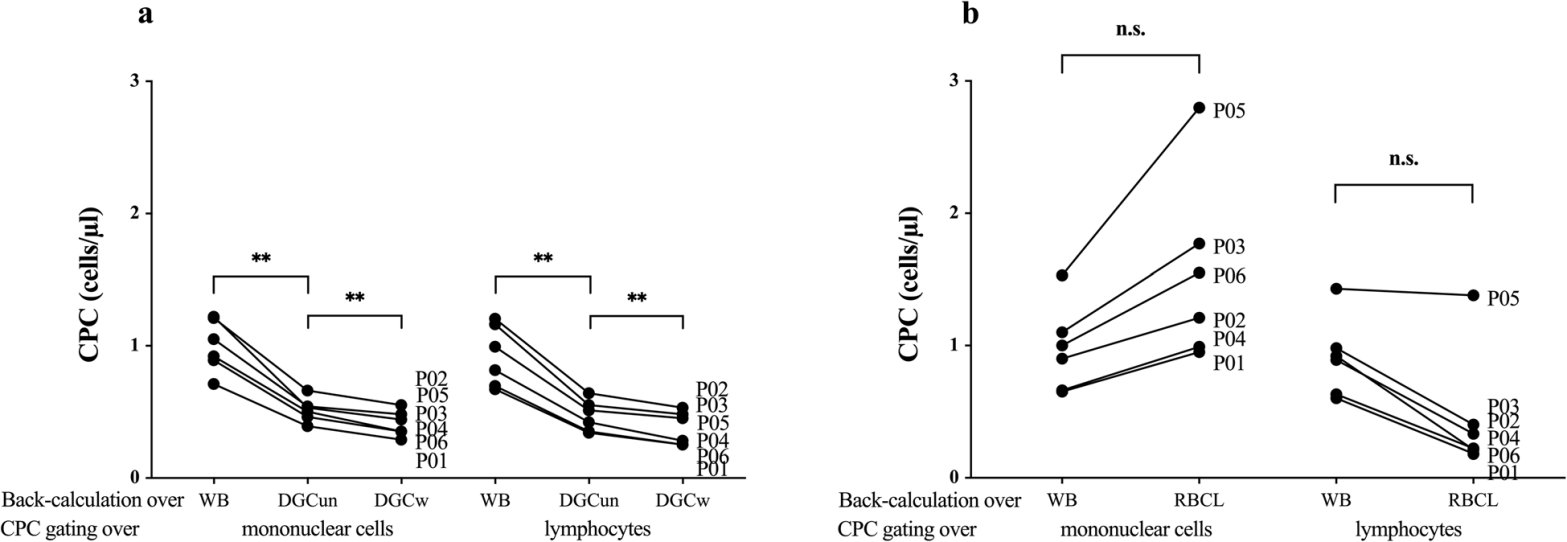
Back calculation results. Back calculation was done by multiplying proportions of gated circulating hematopoietic stem and progenitor cells (CPC) with blood cell counts from either **a** whole blood (WB), directly after density gradient centrifugation (DGCun), and after an additional washing step (DGCw), as well as **b** before (WB) and after the lyse and wash procedure (RBCL). Individual values are displayed. *n* = 6. Repeated-measures ANOVA with Bonferroni post hoc comparisons between all ten different conditions was used for analysis. Significant differences are indicated as follows: **p < 0.01; non-significant, n.s

Back calculation from CPC proportions gated within the LYM region derived from DGC revealed a significant reduction of CPC concentrations back calculated at DGCun (decrease by 50%, *p* = 0.003) as well as from WB to DGCw (decrease by 62%, *p* = 0.001). Results derived from DGCun and DGCw also differed from each other (*p* = 0.007, Fig. 7a). The same was found for back-calculated results from MNC gating, where WB results were significantly different from DGCun (decrease by 48%, *p* = 0.003) and DGCw (decrease by 59%, *p* = 0.001) results and DGCun results also significantly differed from DGCw results (*p* = 0.005, Fig. 7a). Back calculations from CPC proportions gated within the LYM or the MNC region derived from RBCL over WB values were not significantly different from the respective back calculation over blood cell counts measured in RBCL samples (*p* > 0.05, Fig. 7b).

## Discussion

The main result of this study is that values of estimated CPC concentrations do neither depend on the cell isolation technique nor on the gating strategy. However, the accuracy of the values could be improved by correcting according to the change in the LYM/MONO ratio from WB to flow cytometry analysis, i.e., cell change due to mechanical and chemical perturbations during the isolation process should be considered.

LYM and MONO concentrations were similarly affected at DGCun and equally reduced by the following washing step (at DGCw). While the susceptibility of LYM to cell destruction by washing after cell lysis has been reported to be higher compared to MONO [23], DGC has a protecting effect on LYM integrity [20]. Both cell types were lost mostly by DGCun, possibly due to mechanical and chemical destruction. Differences of cell proportions after DGC compared to the respective smear could result from cell apoptosis during DGC since—unlike in a common hemocytometer analysis by light scatter and peroxidase activity—apoptotic cells can easily be excluded during differential smear analysis [29]. RBCL effects on cell concentrations, however, are more difficult to explain. MONO concentrations increased by 682% after RBCL, while more than half of LYM were lost. Neutrophil GRA became more undistinguishable from the MONO subset (lost granularity, lower side scatter, shown in Additional file 4), a fact that Tiirikainen (1995) [22] reported only after additional staining for cell surface antigens. This effect could be due to the chemical perturbation of the lysis solution. MONO proportions being lower and neutrophil GRA (rod-shaped and segmented) proportions being higher on the respective smear than measured by the hemocytometer support this outcome. The inability to correctly distinguish these two cell populations can also be seen as a limitation of the hemocytometer used in this study.

In the detailed analysis of flow cytometry data, it was notable that samples prepared by RBCL showed a higher number of doublets than samples prepared by DGC. Possibly, RBCL by ammonium chloride induced an increase of aggregation dynamics by the binding of neutrophils with remaining platelets [30, 31]. However, this might not be relevant since in rare event analysis such as CPC counting a small number of doublets always remains [32].

Sample preparation steps between DGCw and flow cytometry analysis did not further affect cell content. LYM and MONO proportions measured by flow cytometry were comparable to DGCw. On the contrary, after RBCL, LYM and MONO proportions measured by flow cytometry were significantly higher and lower than in the hemocytometer analysis, respectively. This discrepancy between flow cytometer and hemocytometer analyses in lysed samples was surprising and necessitates further investigation in the future. Possibly, the inversion of MONO and LYM proportions after RBCL from hemocytometer to flow cytometry analysis could be due to the influence of the lysis solution being reversed by the subsequent sample preparation process. In addition, manually adjusting the acquisition gates during flow cytometry supports the correct distinction between LYM and MONO populations, which is not possible during hemocytometer analysis (fixed gates).

Apoptotic LYM proportions were comparable between cell isolation techniques. In contrast, MONO had a higher susceptibility to apoptosis after DGC compared to RBCL. DGC is associated with high levels of stress (1300 rpm) for an extended amount of time (30 min). This could have induced monocyte activation [33] and transiently upregulated the expression of integrins (CD11b/CD18) [34] that were found to be involved in MNC apoptosis [35].

Estimates of CPC concentrations back calculated with WB values were comparable between cell isolation techniques and gating strategies as CPC proportions were similar between procedures and LYM was the largest cell fraction of MNC. In addition, live as well as (early- and late-) apoptotic CPC proportions were comparable among cell isolation techniques and gating strategies. Necrotic CPC proportions did not exist for any of the cell isolation techniques or gating strategies possibly due to rapid cell recovery. The only difference could be found between back-calculated results at the different cell isolation stages of DGC. Estimated CPC concentrations were reduced according to the respective LYM and MONO losses.

The LYM/MONO ratio in the flow cytometry analysis after RBCL was significantly higher (1.7-fold) compared to the WB ratio while LYM/MONO ratio of live cells was even 3.9-fold (DCG) and 2.6-fold (RBCL) increased. This could bias live CPC proportions gated within the MNC region. Therefore, we suggest to correct, e.g., exercise-induced estimated live CPC concentrations by the respective change in LYM/MONO ratio. This mainly plays a role if cells are isolated by DGC since back calculation would be done with WB MNC values.

### Limitations

One technical challenge, and thus, a possible limitation of blood smear, is the chance of confusing a MONO with an atypical LYM [36]. Importantly, in the present study, there were no difficulties in distinguishing MONO from GRA on blood smear.

## Conclusions

In conclusion, estimates of CPC concentrations achieved via back calculation with MONO and/or LYM are similar to WB values, independent of the isolation technique (DGC or RBCL). Results are also independent of the gating strategy. However, one should consider the amount of cell change and apoptosis introduced by mechanical and chemical perturbations during cell isolation and correct final results accordingly (via the change in the LYM/MONO ratio). This is especially important when reporting, e.g., exercise-induced live CPC concentrations after DGC. Since post-exercise CPC increases are small but likely important for regeneration, uncorrected back calculation might bias the regenerative potential in circulation and in turn affect the decision on applications of, e.g., physical exercise as non-invasive therapy.

## Supplementary information

**Additional file 1.** Apoptosis gating of mature blood cells. Aqua vs. Annexin V gating of lymphocytes (a, b), monocytes (c, d) and total mononuclear cells (e, f) after density gradient centrifugation (upper panel) and red blood cell lysis (lower panel). Q1: early-apoptotic cells, Q2: late-apoptotic cells, Q3: necrotic cells, Q4: live cells. Percent numbers indicate cell amount relative to the parent population (%P). (TIFF 7921 kb)

**Additional file 2.** Apoptosis gating of circulating hematopoietic stem and progenitor cells derived from the lymphocyte gate. Aqua vs. Annexin V gating of CD34+/45dim cells derived from the lymphocyte gate as percent of the parent population (% P) after density gradient centrifugation (a) and red blood cell lysis (b). Q1: early-apoptotic cells, Q2: late-apoptotic cells, Q3: necrotic cells, Q4: live cells. (TIFF 8551 kb)

**Additional file 3.** Apoptosis gating of circulating hematopoietic stem and progenitor cells derived from the mononuclear cell gate. Aqua vs. Annexin V gating of CD34+/45dim cells derived from the mononuclear cell gate as percent of the parent population (% P) after density gradient centrifugation (a) and red blood cell lysis (b). Q1: early-apoptotic cells, Q2: late-apoptotic cells, Q3: necrotic cells, Q4: live cells. (TIFF 8784 kb)

**Additional file 4.** Hemocytometer analysis. Hemocytometer (ADVIA 2120i) analysis of whole blood (a) and after red blood cell lysis and an additional wash (RBCL) (b). PEROX, peroxidase channel; BASO, basophil channel; RBC, red blood cells; PLT, platelets; MONO, monocytes; NEU, neutrophils; MN, mononuclear cells; PMN, polymorphonuclear cells; VOL, volume; HC, hemoglobin concentration; CH, channel; VHC, volume/hemoglobin concentration (TIFF 7350 kb)

## Data Availability

All data generated or analyzed during this study are included in this published article [and its supplementary information files].

## Abbreviations

CPC: Circulating hematopoietic stem and progenitor cell(s)
DGC: Density gradient centrifugation
DGCun: DGC unwashed
DGCw: DGC washed
GRA: Granulocyte(s)
LYM: Lymphocyte(s)
MNC: Mononuclear cell(s)
MONO: Monocyte(s)
RBCL: Red blood cell lysis
WB: Whole blood
