# Supplementary figures and images for: Circulating adult stem and progenitor cell numbers—can results be trusted?
Source: Stem Cell Res Ther. 2019 Oct 17;10:305. doi: 10.1186/s13287-019-1403-x (PMC6798345; doi:10.1186/s13287-019-1403-x)

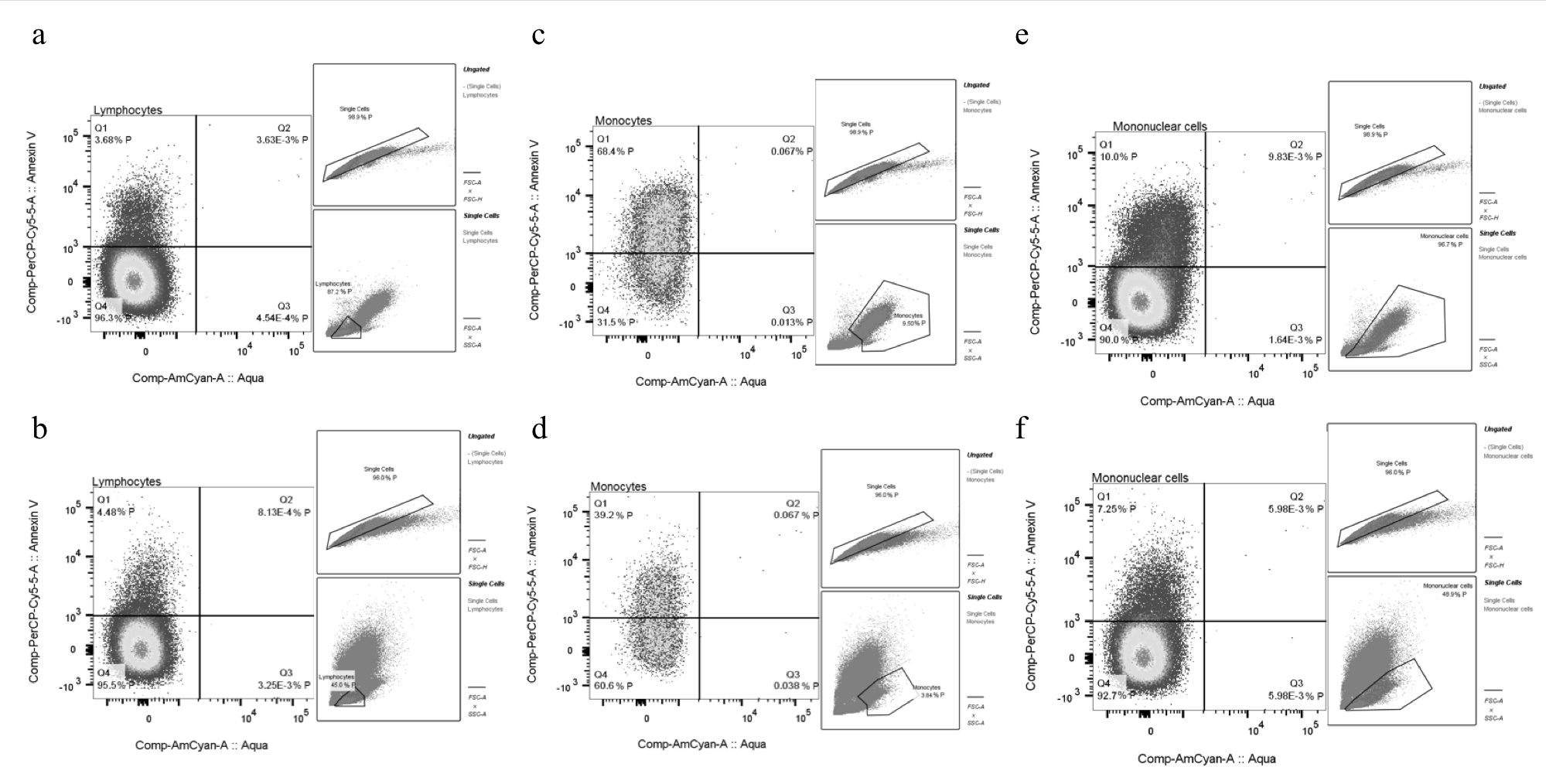

Supplement: Supplementary file 1 — Additional file 1. Apoptosis gating of mature blood cells. Aqua vs. Annexin V gating of lymphocytes (a, b), monocytes (c, d) and total mononuclear cells (e, f) after density gradient centrifugation (upper panel) and red blood cell lysis (lower panel). Q1: early-apoptotic cells, Q2: late-apoptotic cells, Q3: necrotic cells, Q4: live cells. Percent numbers indicate cell amount relative to the parent population (%P). (TIFF 7921 kb) [file 13287_2019_1403_MOESM1_ESM.tiff]

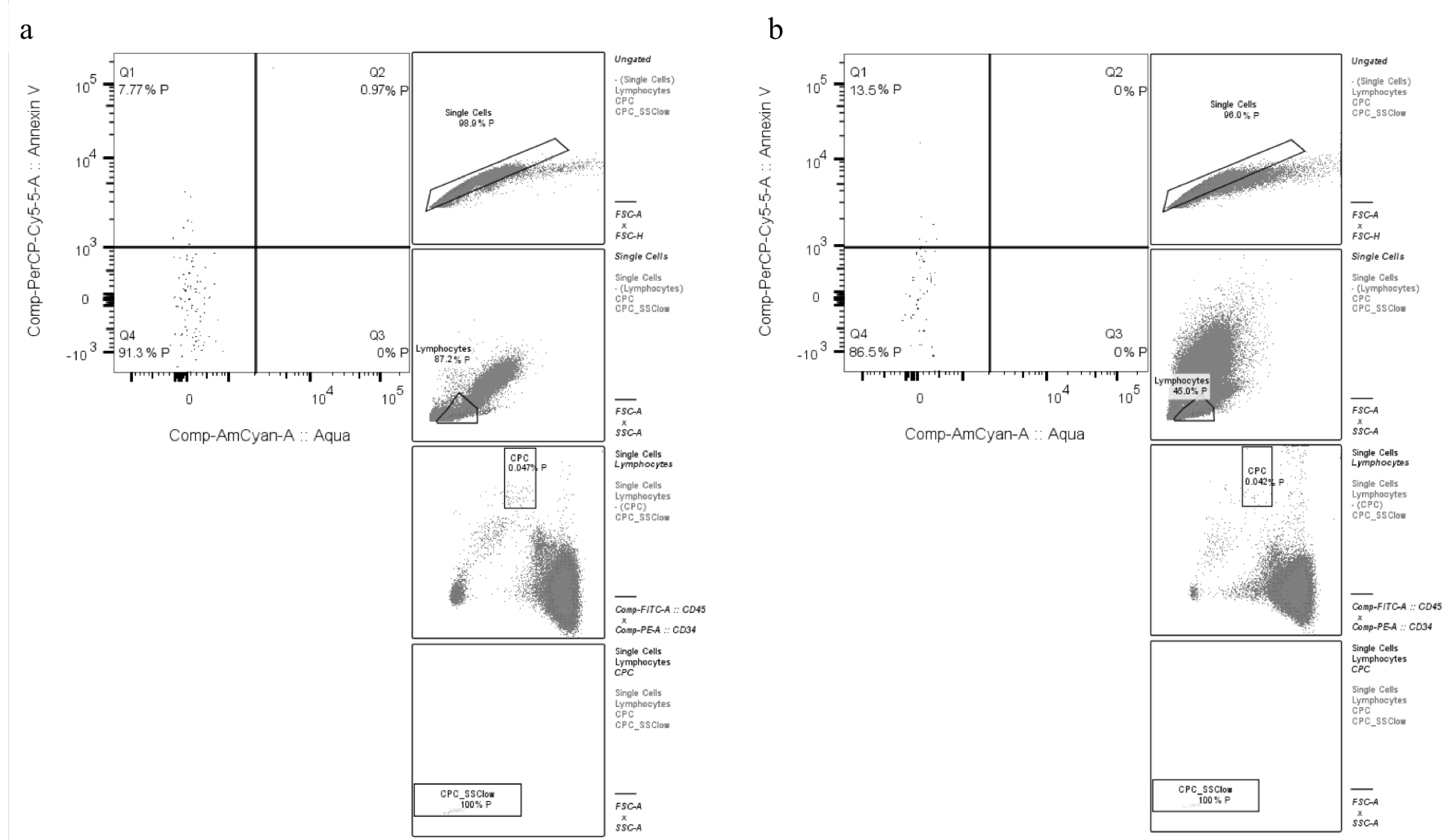

Supplement: Supplementary file 2 — Additional file 2. Apoptosis gating of circulating hematopoietic stem and progenitor cells derived from the lymphocyte gate. Aqua vs. Annexin V gating of CD34+/45dim cells derived from the lymphocyte gate as percent of the parent population (% P) after density gradient centrifugation (a) and red blood cell lysis (b). Q1: early-apoptotic cells, Q2: late-apoptotic cells, Q3: necrotic cells, Q4: live cells. (TIFF 8551 kb) [file 13287_2019_1403_MOESM2_ESM.tiff]

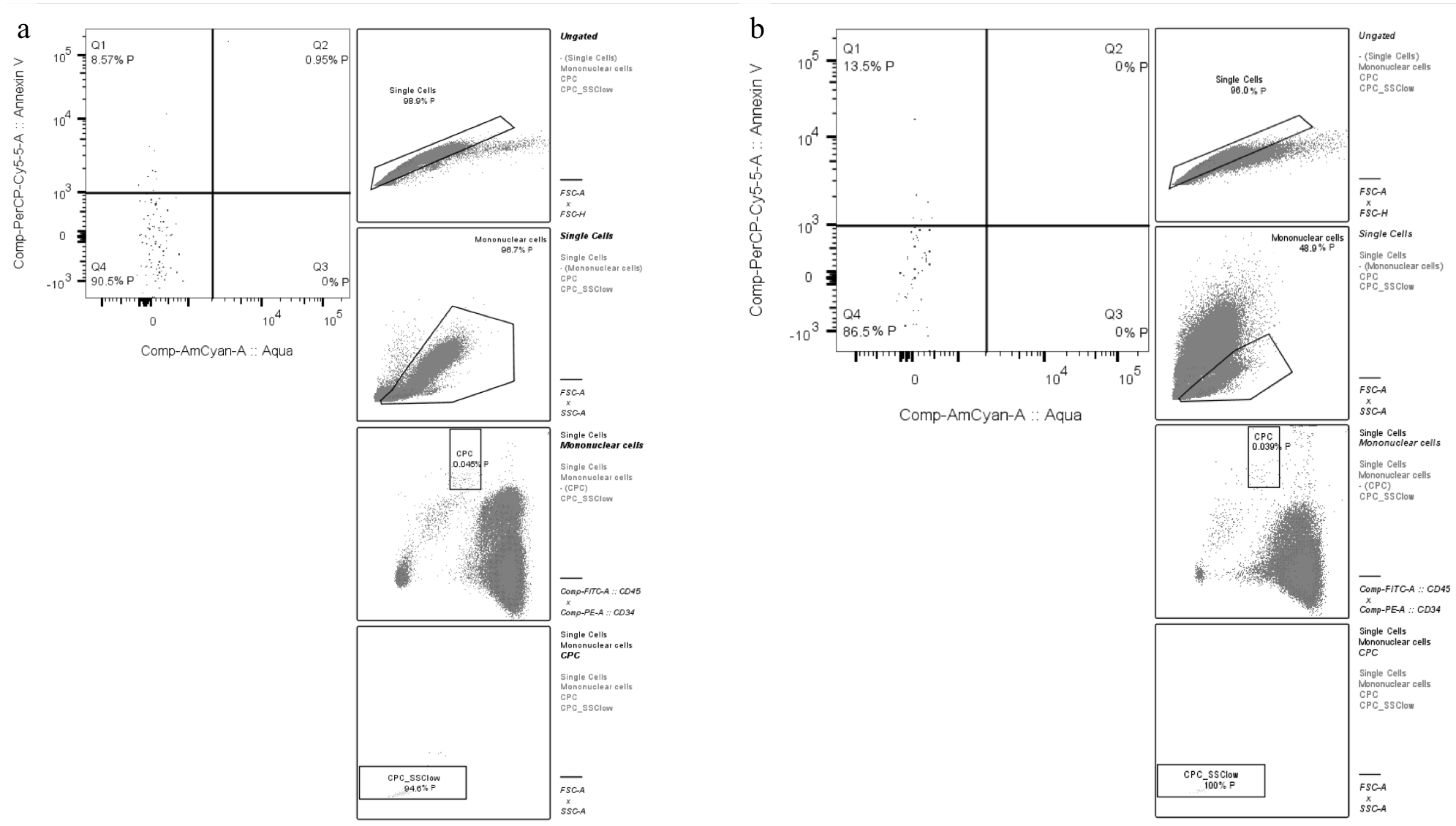

Supplement: Supplementary file 3 — Additional file 3. Apoptosis gating of circulating hematopoietic stem and progenitor cells derived from the mononuclear cell gate. Aqua vs. Annexin V gating of CD34+/45dim cells derived from the mononuclear cell gate as percent of the parent population (% P) after density gradient centrifugation (a) and red blood cell lysis (b). Q1: early-apoptotic cells, Q2: late-apoptotic cells, Q3: necrotic cells, Q4: live cells. (TIFF 8784 kb) [file 13287_2019_1403_MOESM3_ESM.tiff]

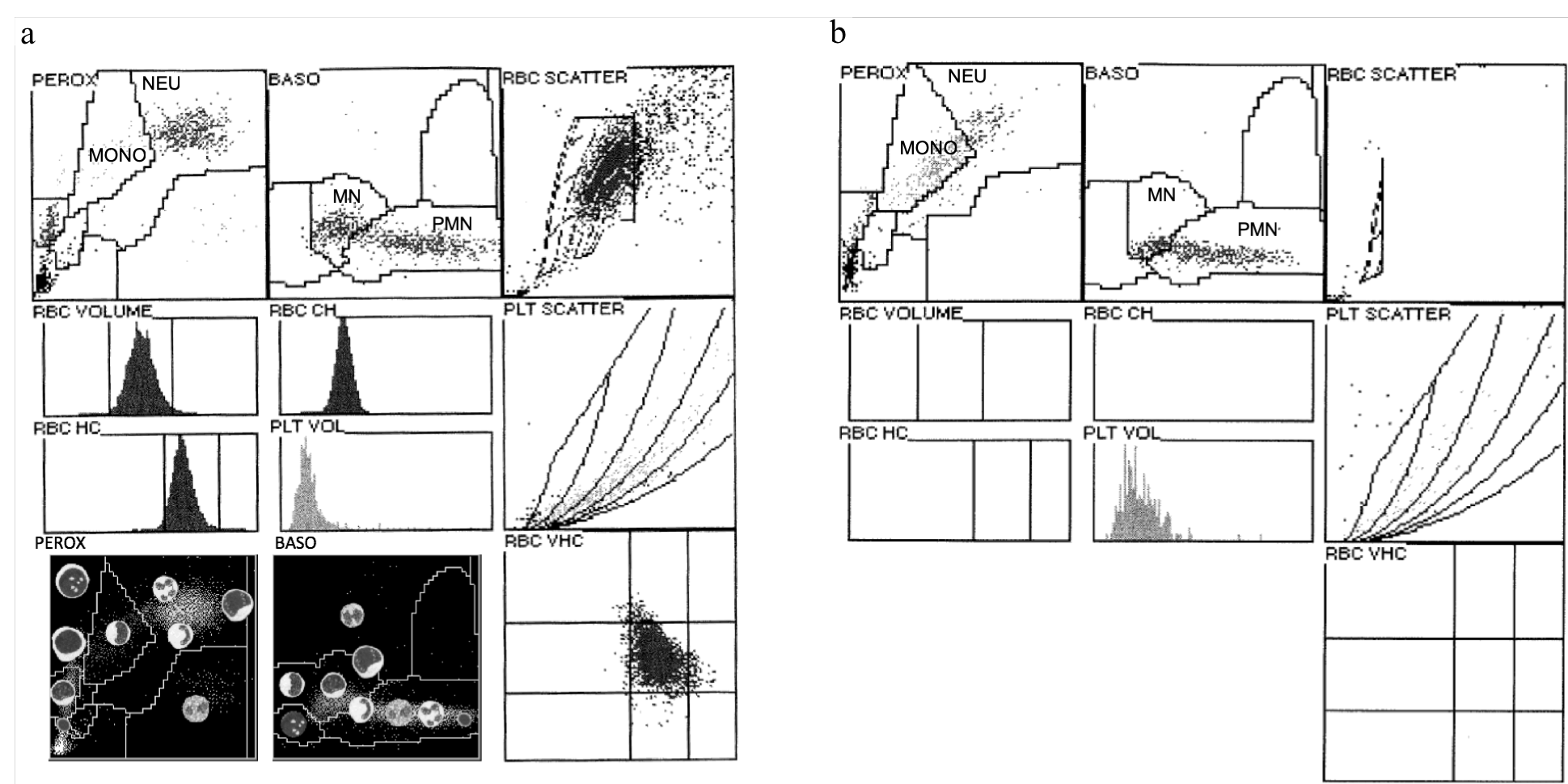

Supplement: Supplementary file 4 — Additional file 4. Hemocytometer analysis. Hemocytometer (ADVIA 2120i) analysis of whole blood (a) and after red blood cell lysis and an additional wash (RBCL) (b). PEROX, peroxidase channel; BASO, basophil channel; RBC, red blood cells; PLT, platelets; MONO, monocytes; NEU, neutrophils; MN, mononuclear cells; PMN, polymorphonuclear cells; VOL, volume; HC, hemoglobin concentration; CH, channel; VHC, volume/hemoglobin concentration (TIFF 7350 kb) [file 13287_2019_1403_MOESM4_ESM.tiff]
